# One-year mortality of patients with ST-Elevation myocardial infarction: Prognostic impact of creatinine-based equations to estimate glomerular filtration rate

**DOI:** 10.1371/journal.pone.0199773

**Published:** 2018-07-06

**Authors:** Yoann Bataille, Olivier Costerousse, Olivier F. Bertrand, Olivier Moranne, Hans Pottel, Pierre Delanaye

**Affiliations:** 1 Quebec Heart-Lung Institute, Quebec, Canada; 2 Department of Cardiology, Centre Hospitalier Régional la Citadelle, Liège, Belgium; 3 Department of Nephrology-Dialysis-Apheresis, CHU de Nîmes, Medical School, University Montpellier-Nimes, Nîmes, France; 4 Department of Public Health and Primary Care, Kulak, University of Leuven, Kortrijk, Belgium; 5 Department of Nephrology, Dialysis, Transplantation, University of Liège (CHU ULg), Liège, Belgium; University of Sao Paulo Medical School, BRAZIL

## Abstract

**Background:**

Renal dysfunction is associated with worse outcomes after primary percutaneous coronary intervention (PCI). However, whether glomerular filtration rate (GFR) estimated with various equations can equally predict outcomes after ST-Elevation Myocardial Infarction (STEMI) is still debated.

**Methods:**

We compared the clinical impact of 3 different creatinine-based equations (Cockcroft and Gault (CG), CKD-epidemiology (CKD-EPI) and Full Age Spectrum (FAS)) to predict 1-year mortality in STEMI patients.

**Results:**

Among 1755 consecutive STEMI patients who had undergone primary PCI included between 2006 and 2011, median estimated GFR was 79 (61;96) with the CG, 81 (65;95) with CKD-EPI and 75 (60;91) mL/min/1.73 m^2^ with FAS equation. Reduced GFR values were independently associated with 1-year mortality risk with the 3 equations. Receiver operating curves (ROC) of CG and FAS equations were significantly superior to the CKD-EPI equation, p = 0.03 and p = 0.01, respectively. Better prediction with FAS and CG equations was confirmed by net reclassification index.

**Conclusions:**

Our results suggest that in STEMI patients who have undergone primary PCI, 1-year mortality is better predicted by CG or FAS equations compared to CKD-EPI.

## Introduction

Chronic Kidney Disease (CKD) is associated with higher early and late mortality risk in many cardiovascular diseases [1–4]. This over-risk has been specifically studied in patients with ST-Elevation Myocardial Infarction (STEMI) both on the short- and long term [5–21]. We have previously demonstrated the impact of CKD in STEMI patients after primary percutaneous coronary intervention (PCI)[22,23]. CKD diagnosis is, however, not so simple and debate still occurs in the literature to know how GFR must be evaluated [24,25]. In daily clinical practice, glomerular filtration rate (GFR) is still the best way to assess renal function. GFR can be measured with high precision by the so-called reference method (inulin, iohexol or iothalamate), but these methods remain relatively cumbersome and are not frequently used in epidemiological studies [26]. Conversely, serum creatinine is easily measured and GFR can be estimated by different equations. The first equation to be used extensively has been the Cockcroft-Gault (CG) equation, proposed in 1976 [27]. Since then, other equations have been developed and more recent data suggest that these equations are more performant to estimate GFR [28–30]. However, the better accuracy of one-given equation to estimate GFR does not necessarily mean that this equation is also better to predict events or mortality. The main objective of this study was to compare the performance of 3 different creatinine-based equations: the CG [27], the CKD-epidemiology (CKD-EPI)[29] and the Full Age Spectrum (FAS)[30]) to predict 1-year mortality in STEMI patients after primary PCI. Because age is a variable in all estimating GFR equations, the second objective of this study was to compare the effect on age on the prediction considering equations but also serum creatinine only.

## Material and methods

The cohort of STEMI patients has been described elsewhere [22,23]. Briefly, the study population included all consecutive 2144 STEMI patients who were referred to our center for primary PCI within 12 hours after symptom onset between 01/2006 and 01/2011. Patients with previous coronary artery bypass graft (n = 73) as well as STEMI patients without significant coronary lesion (n = 51) were excluded from the study. Among the remaining 2020 patients, baseline serum creatinine was available in 1755 patients.

All patients signed an informed consent to be part of a STEMI registry. The registry has been approved by the institution research board of the Quebec Heart-Lung Institute. The inclusion criteria were: chest pain lasting > 30 min, ST-segment elevation ≥ 1mm in ≥ 2 adjacent ECG leads, new left bundle branch block or true posterior MI. All STEMI patients were pre-treated with aspirin, clopidogrel and heparin. Cardiac catheterization was performed via radial approach using 6Fr-guiding catheters in the majority of patients. Adjunctive pharmacotherapy such as bivalirudin or glycoprotein IIb/IIIa inhibitors (GPI) was left at the operators’discretion. If the coronary anatomy was suitable for PCI, the procedure was performed with standard techniques.

The baseline serum creatinine was measured directly on hospital admission in the emergency department or in the catheterization laboratory. Serum creatinine was determined using Jaffe method (Modular P, Roche Diagnostics) and standardized to isotope dilution mass spectrometry [31]. Ability of serum creatinine concentration to predict mortality was tested, and compared to serum creatinine indexed to Q (SCr/Q)[32]. SCr/Q is a variable of the FAS equation and Q is defined as the median SCr value for age-/sex-specific healthy populations [30]. SCr/Q in adults make the concentration independent of gender (as the Q value is not influenced by age in adults, the Q value is 0.7 mg/dL for women, and 0.9 mg/dL for men)[33]. The creatinine-based GFR equations considered are shown in Table 1 [27,29,30]. Both the FAS and CKD-EPI equation give results indexed for body surface area (BSA), therefore the CG had been indexed according to the Gehan equation [34]. Because it is possible that some patients have acute renal dysfunction after STEMI (especially if they were hemodynamically unstable), we used the term “renal dysfunction” instead of “CKD”. Renal dysfunction status was defined by eGFR and 3 stages were considered according to the “Kidney Disease: Improving Global Outcomes”: over 60 mL/min/1.73m^2^ (no renal dysfunction, the reference), between 45 and 60 mL/min/1.73m^2^ and below 45 mL/min/1.73m^2^ [35].

**Table 1:** Creatinine-based equations.

**Cockcroft-Gault (mL/min/1.73m**^**2**^**)**		[(140-age)/(72×SCr)]×weight×(0,85 if woman)x1.73/BSA
**CKD-EPI (mL/min/1.73m**^**2**^**)**	Women	144 x (SCr/0.7)^-0.329^ x 0.993^age^
	SCr≤0.7 mg/dL	
	Women	144 x (SCr/0.7)^-1.209^ x 0.993^age^
	SCr>0.7 mg/dL	
	Men	141 x (SCr/0.9)^-0.411^ x 0.993^age^
	SCr≤0.9 mg/dL	
	Men	141 x (SCr/0.9)^-1.209^ x 0.993^age^
	SCr>0.9 mg/dL	
**FAS (mL/min/1.73m**^**2**^**)**	for 2 ≤ age ≤ 40 years	107.3/(SCr/Q)
	for age > 40 years	107.3/(SCr/Q) x 0.988^(age-40)^

BSA: body surface area, SCr: Serum Creatinine (mg/dL), Q is the median SCr value for age-/sex-specific healthy populations. CKD-EPI: Chronic Kidney Disease-epidemiology; FAS: Full Age Spectrum

Clinical follow-up information was obtained from the referring physicians or by phone contact with patients. Finally, information on vital status at one year of those who were lost for follow-up was collected from the Quebec death registry “Directeur de l’Etat Civil” service counter. The primary outcome was 1-year all-cause mortality and follow-up was completed in 100% of patients.

### Statistical analysis

Data are expressed as mean ± standard deviation (SD) when distribution was normal and as median with interquartile range (IQR) when not. Normality was assessed by the Shapiro-Wilk test. Agreement between equation to classify patients according to CKD stage (above 60 mL/min/1.73m^2^, between 45 and 60 mL/min/1.73m^2^ and below 45 mL/min/1.73m^2^) was tested by Kappa statistics [36].

Cox proportional hazard regressions models were used to study the risk of 1-year mortality associated with renal dysfunction based on the different eGFR equations, where eGFR was used as a categorical or a continuous variable, in non-adjusted models and adjusted for age and gender [37].

Receiver operating curves (ROC curves) (with subgroups based on death) were evaluated from logistic regression models with eGFR, and with eGFR adjusted for age and gender, by comparing the area under the curve [38]. Univariate survival curves (Kaplan-Meier) were calculated for subgroups defined by the fixed eGFR-threshold of 60 mL/min/1.73m^2^ (renal dysfunction versus no renal dysfunction) for the various eGFR-equations [39]. Lastly, net reclassification index (NRI) was also calculated according to the method described by Pencina *et al*.[40,41]. We assumed that a shift to a higher category was an improvement in the non-event group, and a shift to a lower category was an improvement in the event group.

## Results

### Characteristics of the cohort

Baseline clinical and angiographic characteristics of the cohort are summarized in Table 2. In summary, 23.4% of patients were women. Median age was 60 (53;70) years, with 2.3% (n = 41) being younger than 40 years and 37.5% (n = 659) older than 65 years. Median body mass index and body surface area was 26.4 (23.9;29.4) kg/m^2^ and 1.89 (1.75;2.02) m^2^, respectively.

**Table 2:** Baseline clinical, biological, and angiographic characteristics.

Clinical Variables	Patients (n = 1755)
Age, years	62 ± 12
Female, n (%)	411 (23)
BMI (median, IQR)	(26.4, 23.9–29.4)
BSA (median, IQR)	(1.9, 1.7–2.0)
Diabetes mellitus, n (%)	214 (12)
Hypertension, n (%)	757 (43)
Current smoking, n (%)	746 (42)
Hyperlipidemia, n (%)	721 (41)
Prior MI, n (%)	213 (12)
Shock, n (%)	132 (7)
SCr creatinine (median, IQR)	0.96 (0.81;1.12)
eGFR by CG (median, IQR)	79 (61;96)
eGFR by CKD-EPI (median, IQR)	81 (65;95)
eGFR by FAS (median, IQR)	75 (60;91)
Single vessel disease, n (%)	1219 (69)
IRA LM, n (%)	11 (0.6)
IRA LAD, n (%)	703 (40)
IRA RCA, n (%)	793 (45)
IRA CX, n (%)	248 (14)
TIMI flow 0 pre-PCI, n (%) (n = 1752)	1024 (58)
Door to balloon time (n = 1703) (minutes, median, IQR)	40 (29, 69)
TIMI flow 0/1 post-PCI, n (%) (n = 1744)	48 (3)
MBG 0/1, n (%) (n = 1646)	160 (9)
LVEF (%) (n = 1544)	51 ± 13
LVEF <40%, n (%) (n = 1544)	363 (23)
Peak CK-MB (μg/l, median, IQR) (n = 1651)	149 (65, 281)

SCr: serum creatinine concentration; eGFR: estimated glomerular filtration rate; CKD-EPI: CKD-epidemiology; FAS: Full Age Spectrum; BMI: body mass index; BSA: body surface area; MI: myocardial infarction; Shock: end organ hypoperfusion and hemodynamically as systemic systolic pressure < 90 mmHg or systemic systolic pressure ≥ 90 mmHg while using inotropic drugs; SVD: single vessel disease; IRA: infarct-related artery; LM: left main; LAD: left anterior descending artery; RCA: right coronary artery; CX, left circumflex; TIMI: Thrombolysis In Myocardial Infarction; MBG: myocardial blush grade; LVEF: left ventricular ejection fraction; CK-MB: Serum myocardial band of creatine kinase.

Median serum creatinine concentration was 0.96 (0.81;1.12) mg/dL. Median estimated GFR was 79 (61;96), 81 (65;95) and 75 (60;91) mL/min/1.73 m^2^ with the CG, CKD-EPI and FAS equations, respectively. Clinical follow-up was complete for 100% of patients at 1 year (median follow-up: 569 (389;951) days). Cardiologic characteristics of the patients were described in Table 2. The overall 1-year mortality was 8.21%, with the majority of deaths occurring prematurely after STEMI (median 5 (1;29.5) days).

### Renal dysfunction prevalence and mortality according to estimating equations

Prevalence of eGFR below 45 mL/min/1.73m^2^ was 9.3%, 6.8% and 8.8% according to the CG, CKD-EPI and FAS equations, respectively. Concordance was good between CG and CKD-EPI (κ = 0.75, 95% CI: 0.69–0.81) and very good between CG and FAS (κ = 0.87, 95% CI: 0.83–0.91) and between FAS and CKD-EPI (κ = 0.82, 95% CI: 0.77–0.88). The prevalence of eGFR between 45 and 60 mL/min/1.73 m^2^ was 13.8%, 12% and 16.4%. Concordance was moderate between CG and CKD-EPI (κ = 0.45, 95% CI: 0.39–0.52), good between CG and FAS (κ = 0.67, 95% CI: 0.62–0.71) and moderate between FAS and CKD-EPI (κ = 0.58, 95% CI: 0.52–0.63).

### Serum creatinine, equations and mortality at one-year

#### Cox proportional hazard models

**Fig 1:**
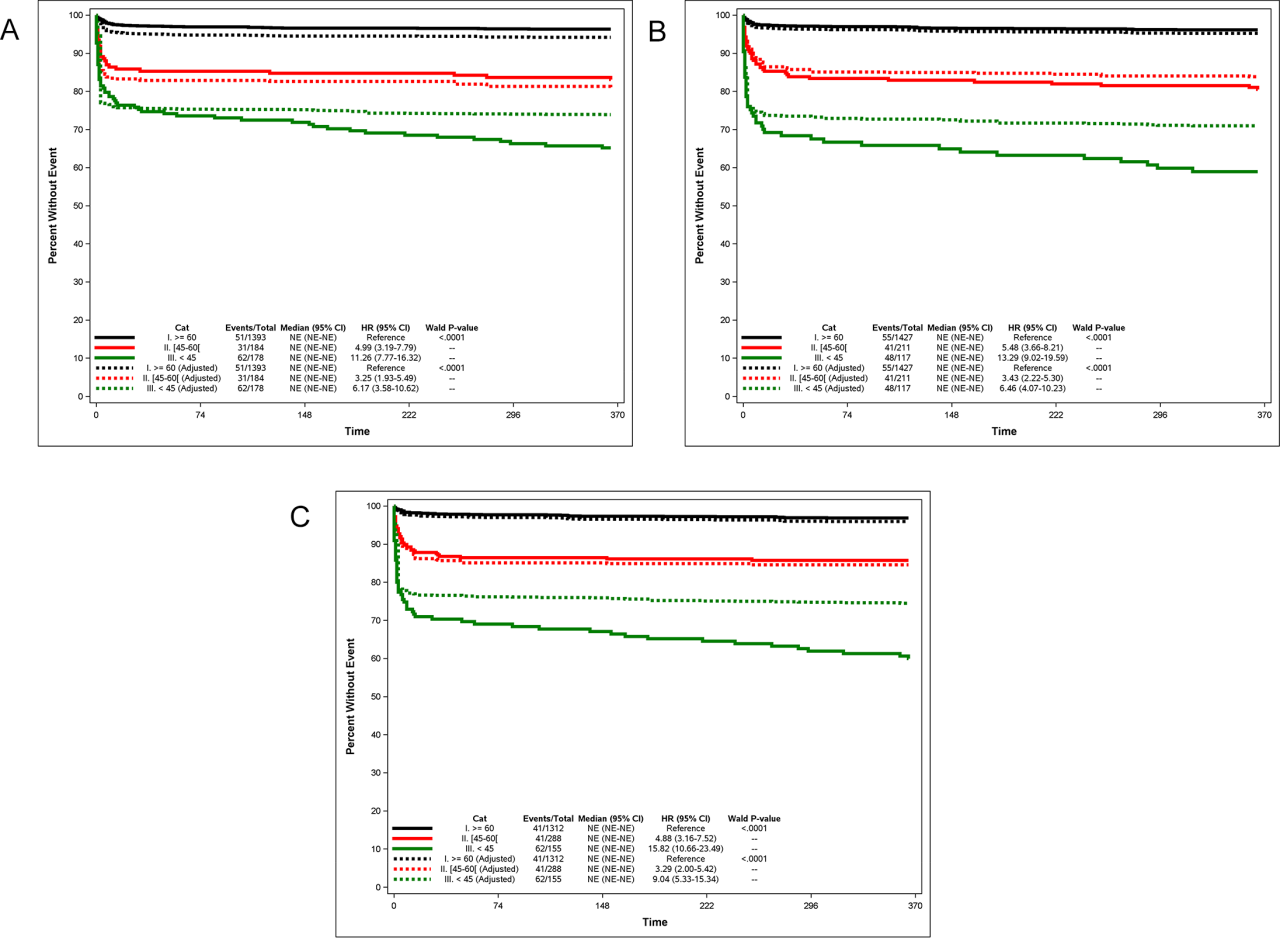
Kaplan-Meier survival curves over one-year in patients with STEMI according to CKD stages and with different equations to estimate GFR. (1A: Cockcroft-Gault, 1B: CKD-EPI and 1C: FAS equation). All survival curves (≥60 mL/min/1.73m^2^, (45-60 (mL/min/1.73m^2^ and <45 mL/min/1.73m^2^) were significantly different from each other, but no significant difference between estimating GFR equations.

We considered 3 groups according to the eGFR: eGFR≥ 60 mL/min/1.73m^2^ (no renal dysfunction, the reference, Group 1), eGFR between 45 and 60 mL/min/1.73m^2^ (Group 2) and eGFR<45 mL/min/1.73m^2^ (Group 3). We used the CG, CKD-EPI and FAS equation to estimate GFR. In the unadjusted analysis, renal dysfunction was highly associated with the mortality risk in group 2: hazard rate (HR): 4.99 95% CI: 3.19–7.79, HR: 5.48 95% CI: 3.66–8.21 and 4HR: .88 95% CI: 3.16–7.52 for the CG, CKD-EPI and FAS equation, respectively. The same observation was made in group 3: HR: 11.26 95% CI: 7.77–16.32, HR: 13.29 95% CI: 9.02–19.59 and HR: 15.82 95% CI: 10.66–23.49 for the CG, CKD-EPI and FAS equations, respectively. Survival curves (and HRs) were significantly different (Logrank test, p<0.0001) between group 1 and groups 2 and 3, but also between group 2 and 3. However, HRs observed were not significantly different between the eGFR equations. After adjustment for age and gender, renal dysfunction remained associated with 1-year mortality: in group 2: HR: 3.25 95% CI: 1.93–5.49 for CG, HR: 3.43 95% CI: 2.22–5.30 for CKD-EPI and HR: 3.29 95% CI: 2.00–5.42 for the FAS equation; in group 3: HR: 6.17 95% CI: 3.58–10.62 for CG, HR: 6.46 95% CI: 4.07–10.23 for CKD-EPI and HR: 9.04 95% CI: 5.33–15.34 for the FAS equation. Survival curves (and HRs) were significantly different (Logrank test, p<0.0001) between group 1 and groups 2 and 3, but also between group 2 and 3 (Fig 1).

However, HRs observed were not significantly different between the eGFR equations. Considering eGFR as a continuous variable, decreased GFR values was also associated with the mortality risk with all 3 equations. By univariate analysis, renal dysfunction (per 10 mL/min/1.73 m^2^ decrease) was related to 1-year mortality by HR 1.62 95% CI: 1.50–1.74 for CG, HR: 1.62 95% CI:1.51–1.74 for CKD-EPI and HR: 1.68 95% CI:1.55–1.81 for FAS equation.

After adjustment renal dysfunction (per 10 mL/min/1.73 m^2^ decrease) remained an independent predictor of 1-year mortality with HR 1.52 95% CI: 1.37–1.68 for CG, HR: 1.46 95% CI:1.34–1.59 for CKD-EPI and HR: 1.68 95% CI: 1.36–1.68 for FAS equation.

#### ROC curves

**Fig 2:**
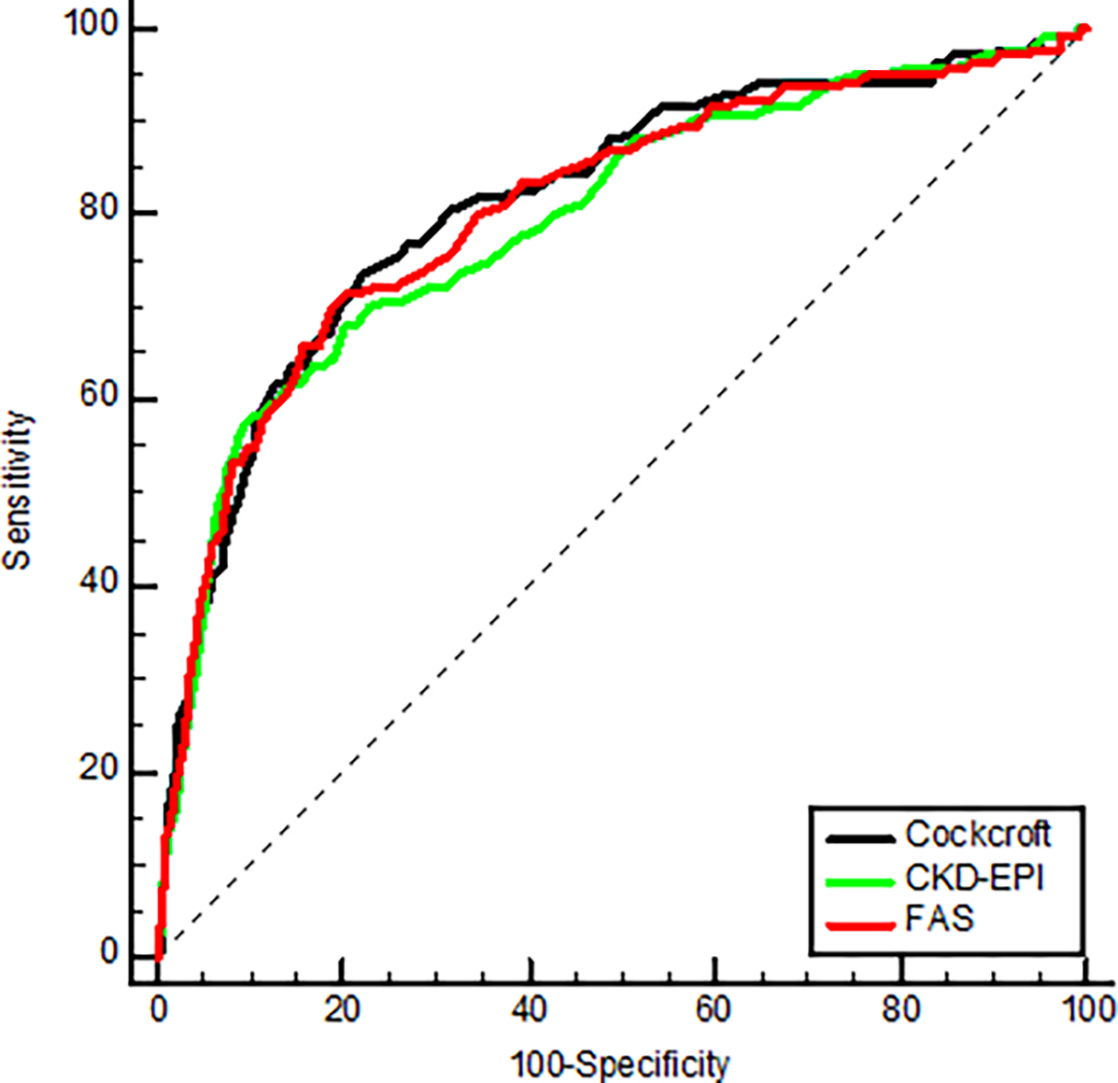
Pairwise comparison of ROC curves to predict one-year mortality with different estimating equations (unadjusted data). No difference between AUC of Cockcroft-Gault and FAS equations but AUC of CKD-EPI equation was significantly lower than Cockcroft-Gault (p = 0.03) and FAS (p = 0.01).

Using the eGFR equations as the only variable in the logistic regression models, the AUCs observed to predict one-year mortality were 0.81 (95% CI: 0.77 to 0.85) for CG, 0.79 (95% CI: 0.75 to 0.84) for CKD-EPI, and 0.805 (95% CI: 0.77 to 0.85) for FAS equation. Pairwise comparison of ROC curves did not show any difference between AUC of CG and FAS equations but AUC of CKD-EPI equation was significantly lower than CG (p = 0.03) and FAS (p = 0.01) (Fig 2). Serum creatinine concentration and SCr/Q also predicted mortality at one-year: 0.72 (95% CI: 0.67 to 0.77) and 0.76 (95% CI: 0.71 to 0.81). AUC observed with SCr/Q was better than AUC of serum creatinine alone (p = 0.0001). However, AUCs of all three equations were significantly higher than AUC of both serum creatinine and SCr/Q (p<0.0001).

Adding age to the model, the AUC observed to predict one-year mortality was 0.813 (95% CI: 0.773 to 0.853), 0.812 (95% CI: 0.774 to 0.851), and 0.809 (95% CI: 0.769 to 0.849) for CG, CKD-EPI and FAS equations respectively. The models smooth off the differences between AUC, resulting in no significant pairwise differences between “age+eGFR” models. The models including age only improved the AUC of the CKD-EPI equation in comparison to the unadjusted model (from 0.794 to 0.812, p = 0.0243). Adding gender next to “age” and “eGFR” in the model did not further improve the AUCs.

Applying age and gender adjustment to serum creatinine and age adjustment SCr/Q impacted the results. AUC of the model including serum creatinine, age and gender was 0.79 (95% CI: 0.75 to 0.83) while AUC for the model with age and SCr/Q was 0.80 (95% CI: 0.76 to 0.84). The 2 models showed the same performance to predict 1-year mortality but the adjusted model for serum creatinine was significantly better than unadjusted (p = 0.04). Moreover, AUCs observed with adjusted models for serum creatinine and SCr/Q were not different from AUCs from adjusted models with the three equations.

#### Net reclassification index (NRI)

NRI analyses are given in Table 3 (CG versus CKD-EPI), Table 4 (CG versus CKD-EPI) and Table 5 (FAS versus CKD-EPI). Analyses showed a worse NRI for CKD-EPI compared to CG (-7.9%, 95% CI: -15.6–0.1%, p = 0.0477), a non-significant NRI for FAS compared to CG (3.5%, 95% CI: -2.2–9.2%, NS) and a better NRI for FAS compared to CKD-EPI (11.7%, 95% CI: 4.5–18.8%, p = 0.0014).

**Table 3:** Reclassification of renal function stages by the CG equation with respect to CKD-EPI equation, and effect of reclassification on mortality risk prediction.

	Non-event							Event				
		CG							CG			
		> = 60	45–60	<45	Total				> = 60	45–60	<45	Total
**CKD-EPI**	> = 60	1263	**103**	**6**	1372		**CKD-EPI**	> = 60	42	**12**	**1**	55
	45–60	**40**	96	**34**	170			45–60	**4**	23	**14**	41
	<45	**0**	**6**	63	69			<45	**0**	**3**	45	48
	Total	1303	205	103	1611			Total	46	38	60	144
		Reclassified by CKDEPI							Reclassified by CKDEPI			
		correct	8,88%						correct	4,86%		
		Incorrect	2,86%						Incorrect	18,75%		
		NRIne	6,02%						NRIe	-13,89%		
					**NRI**	**-7,9%**						
					**SE**	**4,0%**						
					**95%CI**	**[-1.6%,0.0%]**						
					**p**	**0,0477**						

CG: Cockcroft-Gault; CKD-EPI: Chronic Kidney Disease-epidemiology

**Table 4:** Reclassification of renal function stages by the FAS equation with respect to CG equation, and effect of reclassification on mortality risk prediction.

	Non-event							Event				
		CG							CG			
		> = 60	45–60	<45	Total				> = 60	45–60	<45	Total
**FAS**	> = 60	1235	**36**	**0**	1271		**FAS**	> = 60	39	**2**	**0**	41
	45–60	**68**	159	**20**	247			45–60	**7**	31	**3**	41
	<45	**0**	**10**	83	93			<45	**0**	**5**	57	62
	Total	1303	205	103	1611			Total	46	38	60	144
		Reclassified by FAS							Reclassified by FAS			
		correct	3,48%						correct	8,33%		
		Incorrect	4,84%						Incorrect	3,47%		
		NRIne	-1,37%						NRIe	4,86%		
					**NRI**	**3,5%**						
					**SE**	**2,9%**						
					**95%CI**	**[-0.2%,0.9%]**						
					**p**	**0,2321**						

CG: Cockcroft-Gault; FAS: Full Age Spectrum

**Table 5:** Reclassification of renal function stages by the FAS equation with respect to CKD-EPI equation, and effect of reclassification on mortality risk prediction.

	Non-event							Event				
		CKD-EPI							CKD-EPI			
		> = 60	45–60	<45	Total				> = 60	45–60	<45	Total
**FAS**	> = 60	1260	**11**	**0**	1271		**FAS**	> = 60	41	**0**	**0**	41
	45–60	**112**	134	**1**	247			45–60	**14**	25	**2**	41
	<45	**0**	**25**	68	93			<45	**0**	**16**	46	62
	Total	1372	170	69	1611			Total	55	41	48	144
		Reclassified by FAS							Reclassified by FAS			
		correct	0,74%						correct	20,83%		
		Incorrect	8,50%						Incorrect	1,39%		
		NRIne	-7,76%						NRIe	19,44%		
					**NRI**	**11,7%**						
					**SE**	**3,7%**						
					**95%CI**	**[0.5%,1.9%]**						
					**p**	**0,00140**						

Chronic Kidney Disease-epidemiology; FAS: Full Age Spectrum

## Discussion

In the current analysis, we showed that the CG and FAS equations were better predictors of mortality than the recommended CKD-EPI formula, with better NRI and AUC. As expected, the proportion of women admitted for STEMI is concordant with the current literature, as cardiovascular disease develops 7 to 10 years later in women than in men. It is assumed that exposure to endogenous estrogens during the fertile period of life delays the manifestation of atherosclerotic disease in women [42]. We also showed that age is, as expected, an important variable for mortality prediction. Indeed, in ROC curve analyses, the difference in AUC between equations disappeared when age was considered in the model. Still more illustrative, the better prediction using creatinine-based equations (all including the age variable) over serum creatinine alone (or scaled creatinine) totally disappears when age is included in the model. Also, in Cox regression analyses, we showed that age strongly impacts mortality risk in the lower GFR group (<45 mL/min/1.73m^2^), which is not unexpected given the physiological decline of GFR with aging [43,44]. Accordingly, a GFR below 45 mL/min/1.73m^2^ means a much larger relative decline in young patients than in the elderly for who a GFR between 45 and 60 mL/min/1.73m^2^ could even been considered as normal [45,46].

There are several ways to estimate GFR as different creatinine-based equations have been developed and these equations have potentially different impacts on the mortality risk [1,5,6,8,10,11,13,14,20,21]. Our data showed that the predictive argument, i.e. the potential better ability of a given equation to predict mortality or other outcomes, to favor for one equation over another, may be spurious. As an example, the promotors of the CKD-EPI equation, an equation published in 2009 by Levey et al, frequently argue that CKD-EPI equation is a better risk prediction factor than Modified Diet in Renal Disease (MDRD) study equation [1,5,8,14], which was published by the same group in 1999 and became first equation to be more popular than the CG equation [47]. However, these authors have not tested other equations, such as the old CG equation in comparison with the MDRD or CKD-EPI equations. When compared to these two equations, the CG equation has been shown to be better predictive of mortality in patients with STEMI or ischemic heart disease [10–12,20,21], but also in other cardiologic populations [48–54]. On the contrary, Orvin et al showed a slightly better performance of the MDRD than CG to predict mortality at one-year after acute coronary syndrome [13]. However, the difference was not significant between the CG and CKD-EPI equations. Similar predictive values of CG and CKD-EPI were also shown in two other cohorts of patients with heart failure [55,56].

As suggested by some authors and supported by our own data, the ability of one equation to better predict mortality could be due to the mathematical exponent applied to age in the equation [11]. Having said that, one can though discuss the balance of each equation in their ability to predict the risk on one side and estimate GFR (in comparison to true measured GFR) on the other side. If CG is doing well in predicting the risk, data from the literature have clearly shown that its performance is less good than other recent equations to estimate GFR, especially with a lack of precision [28,57]. The CKD-EPI equation does not seem a superior equation to predict the risk in our cohorts, and in others [10–12,20,21]. Eventually, the FAS equation could be considered as the best balance between GFR estimation, equivalent to CKD-EPI in adults and even better in the elderly, and risk mortality, equivalent to CG [25,30,58].

There are limitations to our work. In all creatinine-based equations, the most important variable is serum creatinine and this biomarker is dependent on non-GFR determinants, the most important being muscle mass [59]. Measured GFR by a reference method was not available in this cohort [60]. Moreover, cystatin C, another GFR biomarker, was not available while this biomarker was shown to better predict mortality and morbidity [2,5,7,9,14]. However, the added value of cystatin C as a predictor could be explained by non-GFR determinants, such as inflammation [61]. Also, in our cohort, data on the evolution of renal function in the days after STEMI and PCI are unfortunately not available. Another limitation is the fact that eGFR assessment was based on a single serum creatinine measurement at the time of hospital admission. This measurement could have been affected by hemodynamic instability in some patients. In other words, it is possible that part of decreased GFR results are due to acute kidney dysfunction, and not CKD. We do not have complete data on hemodynamic stability at admission, except that 7.5% of patients were considered in cardiogenic shock. As expected, the percentage of patients in cardiogenic shock was higher in those with low eGFR (25.2% in the patients with eGFR by FAS below 45 mL/min/1.73m^2^; 13.9% in the patients with eGFR by FAS between 45 and 60 mL/min/1.73m^2^; 4% in the patients with eGFR by FAS above 60 mL/min/1.73m^2^). Again, this limitation is shared by all studies on the topic. Also in clinical practice, “true” baseline serum creatinine is not known at admission. The last limitation was that patients included here were all Caucasians and our results might not be directly applicable to other ethnicities. Finally, the goal of our analysis was to study the impact of the choice of eGFR equation on the prognosis of STEMI patients, not on the effect of renal dysfunction *per se*, as this point has been previously published [23]. However, we have added some adjusted results in supplements (S1 File).

In conclusion, we showed that renal dysfunction is associated with one-year mortality in our cohort of patients with STEMI. Beyond serum creatinine and whatever creatinine-based equation, age is an important variable that could explain the superiority of one equation over another in its ability to predict the risk. Our results suggest that in STEMI patients who have undergone primary PCI, 1-year mortality is better associated by CG or FAS equations compared to CKD-EPI.

## Supporting information

S1 File FAS equation and association with mortality in a multivariate model. (DOCX)
