# Supplementary material for: One-year mortality of patients with ST-Elevation myocardial infarction: Prognostic impact of creatinine-based equations to estimate glomerular filtration rate
Source: PLoS One. 2018 Jul 6;13(7):e0199773. doi: 10.1371/journal.pone.0199773 (PMC6034802; doi:10.1371/journal.pone.0199773)
Supplement: S1 File — (DOCX) [file pone.0199773.s001.docx]

**S1 File: FAS equation and association with mortality in a multivariate model**

Age is without doubt one of the main variables influencing outcomes in STEMI. Because age and gender are variables of eGFR equations and the goal of the present analysis is to compare the impact of different eGFR equations in cardiovascular disease risk models, we presented unadjusted and age/gender adjusted Cox models. However, other clinical variables could also influence the outcome. Such variables are described in Table S1. This table shows the patient characteristics that might have an impact on mortality, per eGFR-category. We based this table on the FAS-equation. We then calculated the Cox model, adjusting for those covariates that were significantly different between the eGFR-categories. To make a fair comparison between the unadjusted model, the model adjusted only for age and gender, and the model adjusted for the significant variables in the eGFR-categories table, we only included those covariates that did not change the number of events or total number of patients, that is, we excluded the covariates that had missing values: TIMI Flow 0 pre-PCI %, TIMI Flow 0 post-PCI %, Door to ballon time, MBG 0/1 % and LEVF. Results are shown in Table S2: including other variables had an effect on the hazard ratios (the HRs become smaller), but they remain significantly different from ‘1’. The reduction of the HR is mainly in category III (< 45), but the main message that eGFR-categorization has an important effect on the mortality risk remains, even after adjusting for many variables that are known to impact mortality in STEMI patients.

Table S1: Patient Characteristics according to FAS categories

|  | **Total**  **(n=1755)** | **FAS<45 mL/min/1.73m²**  **(n=155)** | **FAS between 45 and 60 mL/min/1.73m²**  **(n=288)** | **FAS>60 mL/min/1.73m²**  **(n=1312)** | **p-value** |
| --- | --- | --- | --- | --- | --- |
| **Age** | 62±12 | 78±10 | 72±10 | 58±10 | < 0.0001 |
| **Men %** | 77 | 53 | 64 | 82 | < 0.0001 |
| **BMI** | 26 (24 ; 29) | 25 (23 ; 28) | 26 (24 ; 29) | 27 (24 ; 30) | < 0.0001 |
| **BSA** | 1.89 (1.75 ; 2.02) | 1.77 (1.68 ; 1.9) | 1.85 (1.66 ; 2.00) | 1.91 (1.79 ; 2.04) | < 0.0001 |
| **Diabetes %** | 12.2 | 23.2 | 17.0 | 9.8 | < 0.0001 |
| **Hypertension %** | 43.1 | 72.9 | 60.4 | 35.8 | < 0.0001 |
| **Current Smoking %** | 42.5 | 22.6 | 27.1 | 48.2 | < 0.0001 |
| **Hyperlipemia %** | 41.1 | 51.6 | 44.4 | 39.1 | 0.0050 |
| **Prior MI %** | 12.1 | 24.5 | 12.5 | 10.6 | < 0.0001 |
| **Shock %** | 7.5 | 25.2 | 13.9 | 4.0 | < 0.0001 |
| **Single Vessel Disease %** | 69.5 | 47.7 | 59.7 | 74.2 | < 0.0001 |
| **IRA LM %** | 0.6 | 3.2 | 0.7 | 0.3 | 0.0011 |
| **IRA LAD %** | 40.1 | 38.7 | 36.1 | 41.2 | 0.2782 |
| **IRA CX %** | 14.1 | 11.0 | 12.5 | 14.9 | 0.2847 |
| **IRA RCD %** | 45.2 | 47.1 | 50.7 | 43.8 | 0.0903 |
| **TIMI Flow 0 pre-PCI %** | 58.4  (n=1752) | 48.4  (n=155) | 56.3  (n=286) | 60.1  (n=1311) | 0.0141 |
| **Door to ballon time** | 40 (29 ; 69)  (n=1703) | 45 (32 ; 77)  (n=148) | 45 (31 ; 75)  (n=281) | 38 (28 ; 66)  (n=1274) | < 0.0001 |
| **TIMI Flow 0 post-PCI %** | 2.8  (n=1744) | 7.1  (n=155) | 5.6  (n=288) | 1.6  (n=1301) | < 0.0001 |
| **MBG 0/1 %** | 9.7  (n=1646) | 18.5  (n=146) | 14.6  (n=274) | 7.6  (n=1226) | < 0.0001 |
| **LEVF** | 51±14  (n=1544) | 44±16  (n=128) | 49±16  (n=251) | 52±13  (n=1165) | < 0.0001 |
| **LEVF<40% %** | 23.5  (n=1544) | 45.3  (n=128) | 28.7  (n=251) | 20.0  (n=1165) | < 0.0001 |
| **Peak CK-MB** | 149 (66 ; 281)  (n=1651) | 149 (61 ; 268)  (n=139) | 139 (67 ; 285)  (n=270) | 152 (66 ; 281)  (n=1242) | 0.9079 |

p-values are obtained by anova or kruskal-wallis for continuous data and by the exact chi-square test for categorical data

Table S2: Cox proportional hazard regression models for mortality, stratified by eGFR-category

|  | Unadjusted | | Adjusted (for age/sex) | | Adjusted for all covariates | |
| --- | --- | --- | --- | --- | --- | --- |
| eGFR-equation | HR (I vs II) | HR (I vs III) | HR (I vs II) | HR (I vs III) | HR (I vs II) | HR (I vs III) |
| FAS | 4.88 [3.16-7.52] | 15.82 [10.66-23.49] | 3.29 [2.00-5.42] | 9.04 [5.33-15.34] | 3.23 [1.96-5.32] | 7.91 [4.64-13.48] |
| CKD-EPI | 5.48 [3.66-8.21] | 13.29 [9.02-19.59] | 3.43 [2.22-5.30] | 6.46 [4.07-10.23] | 3.44 [2.21-5.34] | 5.84 [3.66-9.34] |
| Cockcroft-Gault | 4.91 [3.19-7.54] | 13.09 [8.91-19.24] | 3.26 [1.97-5.39] | 7.16 [4.15-12.36] | 3.16 [1.89-5.26] | 5.96 [3.42-10.41] |

HR = Hazard Ratio; I = FAS > 60mL/min/1.73m² (used as the reference group) ; II = FAS between 45 and 60mL/min/1.73m² and III = FAS < 45mL/min/1.73m².
